# Immune inflammation indicators and implication for immune modulation strategies in advanced hepatocellular carcinoma patients receiving sorafenib

**DOI:** 10.18632/oncotarget.11565

**Published:** 2016-08-24

**Authors:** Andrea Casadei Gardini, Emanuela Scarpi, Luca Faloppi, Mario Scartozzi, Nicola Silvestris, Daniele Santini, Giorgio de Stefano, Giorgia Marisi, Francesca V. Negri, Francesco Giuseppe Foschi, Martina Valgiusti, Giorgio Ercolani, Giovanni Luca Frassineti

**Affiliations:** ^1^ Department of Medical Oncology, Istituto Scientifico Romagnolo per lo Studio e Cura dei Tumori (IRST) IRCCS, Meldola, Italy; ^2^ Unit of Biostatistics and Clinical Trials, IRST IRCCS, Meldola, Italy; ^3^ Department of Medical Oncology, Ospedale Generale Provinciale di Macerata ASUR Marche AV3, Macerata, Italy; ^4^ Department of Medical Oncology, University Hospital Cagliari, Cagliari, Italy; ^5^ Medical Oncology Unit, Cancer Institute “Giovanni Paolo II”, Bari, Italy; ^6^ Medical Oncology Department, University Campus Bio-Medico, Via Álvaro del Portillo, Rome, Italy; ^7^ Infectious Diseases and Interventional Ultrasound Unit, D. Cotugno Hospital, Naples, Italy; ^8^ Biosciences Laboratory, IRST IRCCS, Meldola, Italy; ^9^ Medical Oncology Unit, University Hospital, Parma, Italy; ^10^ DPT Internal Medicine, Faenza Hospital, Faenza, AUSL Romagna, Forli, Italy; ^11^ Department of General Surgery, Morgagni-Pierantoni Hospiatal, AUSL Romagna, Forli, Italy; ^12^ Department of Medical and Surgical Sciences, University of Bologna, Bologna, Italy

**Keywords:** systemic immune-inflammation index, inflammation, biomarker, hepatocellular carcinoma, neutrophil-to-lymphocyte ratio

## Abstract

We evalueted a systemic immune-inflammation index (SII), neutrophil-to-lymphocyte ratio (NLR) and platelet-lymphocyte ratio (PLR) with the aim to explored their prognostic value in patients with advanced hepatocellular carcinoma (HCC) treated with sorafenib. 56 advanced HCC patients receiving sorafenib were available for our analysis. Lymphocyte, neutrophil and platelet were measured before beginning of treatment and after one month. Patient with SII ≥ 360 showed lower median PFS (2.6 *vs.* 3.9 months, *P* < 0.026) and OS (5.6 *vs.* 13.9 months, *P* = 0.027) with respect to patients with SII < 360.

NLR ≥ 3 had a lower median PFS (2.6 *vs.* 3.3 months, *P* < 0.049) but not OS (5.6 *vs.* 13.9 months, *P* = 0.062) than those with NLR < 3. After adjusting for clinical covariates SII and NLR remained an independent prognostic factor for OS. The SII and NLR represent potential prognostic indicator in patients with advanced HCC treated with sorafenib.

## INTRODUCTION

Hepatocellular carcinoma (HCC) represents the most common primary liver cancer with an increasing incidence [1].

The introduction of Sorafenib, currently representing the standard of care of advanced HCC, changed the clinical landscape even if a large proportion of patients show a limited efficacy with respect to toxic effects [2, 3, 4, 5, 6, 7]. Until now predictive biomarkers of sorafenib efficacy or resistance have yet to be identified [8, 9, 10, 11, 12, 13].

Systemic inflammatory responses have been shown to reflect the promotion of angiogenesis, DNA damage and tumor invasion through up-regulation of cytokines [14]. Previous research revealed that lymphocytes play a crucial role in tumor defense by inducing cytotoxic cell death and inhibiting tumor cell proliferation and migration [15]. In consideration of these factors, several inflammation and immune-based prognostic scores, such as lymphocyte count, neutrophil-lymphocyte ratio (NLR), and systemic immune-inflammation index (SII), have been developed to predict survival and recurrence in cancers, including HCC [16, 17].

Cancer immunotherapy has made huge progress in the last few years. In particular, recent studies focalize the role of immune system in HCC. In fact, the unique immune response in the liver favors tolerance, which can represent a genuine challenge for conventional immunotherapy in patients with HCC [18].

Herein, we evaluated the potential role of SII, NLR and PLR as predictors of outcome in HCC patients treated with sorafenib.

## RESULTS

### Patient characteristics

56 patients diagnosed with HCC were consecutively treated with sorafenib. The patients caracteristics and clinical outcome show in Table 1.

**Table 1:** Univariate analysis of progression-free survival (PFS) and overall survival (OS)

PFS	No. patients (%)	No. events	Median PFS (95% CI)	*P*	HR (95% CI)	*P*
Overall	56 (100)	46	2.8 (2.6-3.9)	-	-	-
Age, years (continuous variable)	-	-	-	-	0.99 (0.96-1.01)	0.286
Gender						
Male	47 (83.9)	39	2.6 (2.2-2.9)		1.00	
Female	9 (16.1)	7	8.5 (5.2-18.8)	0.018	0.37 (0.16-0.87)	0.022
Etiology						
Other	25 (44.6)	19	2.9 (1.4-6.0)		1.00	
Viral	31 (55.4)	27	2.8 (2.2-5.2)	0.686	1.13 (0.62-2.07)	0.687
ECOG PS						
0	31 (55.4)	26	3.9 (2.5-8.2)		1.00	
≥1	25 (44.6)	20	2.6 (1.8-2.9)	0.170	1.53 (0.83-2.81)	0.175
BCLC stage						
B	13 (23.2)	10	6.0 (1.4-18.8)		1.00	
C	43 (76.8)	36	2.7 (2.3-3.3)	0.060	2.01 (0.96-4.20)	0.065
Alpha-fetoprotein:						
<400	32 (59.3)	24	2.7 (2.0-3.9)		1.00	
≥400	22 (40.7)	20	3.7 (2.3-10.8)	0.123	0.59 (0.30-1.17)	0.128
MELD score						
≤10	44 (78.6)	37	2.8 (2.6-5.2)		1.00	
>10	12 (21.4)	9	2.6 (0.9-3.7)	0.832	1.09 (0.50-2.36)	0.833
Extrahepatic spread						
Yes	16	14	2.6 (1.8-3.9)		1.00	
No	40	32	3.3 (2.6-6.0)	0.110	0.59 (0.31-1.14)	0.116

OS	No. patients (%)	No. events	Median OS (95% CI)	*P*	HR (95% CI)	*P*
Overall	56 (100)	39	6.9 (5.2-14.6)	-	-	-
Age, years (continuous variable)	-	-	-	-	0.98 (0.96-1.00)	0.107
Gender						
Male	47 (83.9)	32	6.7 (3.7-13.9)		1.00	
Female	9 (16.1)	7	19.0 (5.7-23.0)	0.221	0.60 (0.26-1.38)	0.226
Etiology						
Other	25 (44.6)	15	6.8 (3.7-14.6)		1.00	
Viral	31 (55.4)	24	6.9 (3.9-15.6)	0.619	1.18 (0.61-2.30)	0.620
ECOG PS						
0	31 (55.4)	24	13.9 (3.7-15.6)		1.00	
≥1	25 (44.6)	15	6.7 (3.2-22.8)	0.794	0.91 (0.47-1.79)	0.794
BCLC stage						
B	13 (23.2)	8	15.8 (1.4-24.0)		1.00	
C	43 (76.8)	31	6.7 (3.9-13.9)	0.092	1.96 (0.88-4.36)	0.098
Alpha-fetoprotein						
<400	32 (59.3)	19	6.7 (4.5-22.8)		1.00	
≥400	22 (40.7)	18	11.2 (3.2-15.8)	0.762	1.11 (0.57-2.13)	0.761
MELD score						
≤10	44 (78.6)	31	11.2 (5.2-15.6)		1.00	
>10	12 (21.4)	8	6.7 (0.9-34.2)	0.696	1.17 (0.53-2.59)	0.697
Extrahepatic spread						
Yes	16	13	5.2 (3.1-14.6)		1.00	
No	40	26	10.4 (5.7-15.6)	0.257	0.68 (0.34-1.33)	0.260

### SII, NLR PLR and clinical outcome

**Figure 1:**
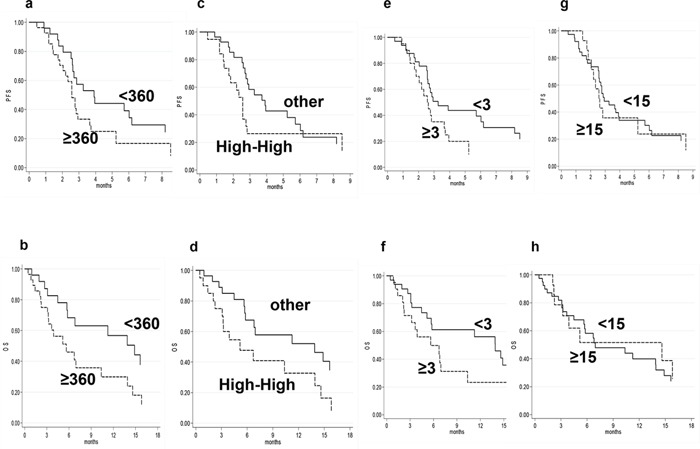
**a.** Progression-free survival (PFS) in relation to SII at baseline; **b.** Overall survival (OS) in relation to SII at baseline; **c.** Progression-free survival (PFS) in relation to SII modifications during the course of treatment; **d.** Overall survival (OS) in relation to SII modifications during the course of treatment; **e.** Progression-free survival (PFS) in relation to NLR; **f.** Overall survival (OS) in relation to NLR; **g.** Progression-free survival (PFS) in relation to PLR; h. Overall survival (OS) in relation to PLR.

SII ≥ 360 at baseline was associated with a median PFS of 2.6 months (95% CI 2.0-2.9) compared to 3.9 months (95% CI 2.8-6.2) for patients with SII < 360 (*P* = .026) (HR 2.01, 95%CI 1.07-3.75, *p* = 0.029) (Figure 1a). SII ≥ 360 was associated with a median OS of 5.6 months (95% CI 3.2-10.4) compared to 13.9 months (95% CI 5.7-22.8) for patients with SII < 360 (*P* = .024) (HR 2.13, 95%CI 1.09-4.17, *p* = 0.027) (Figure 1b).

SII ≥ 360 at 1 months was associated with a median PFS of 2.6 months (95% CI 1.8-3.3) compared to 3.9 months (95% CI 2.8-6.2) for patients with SII < 360 (*P* = .024) (HR 2.00, 95%CI 1.08-3.70, *p* = 0.027). SII ≥ 360 was associated with a median OS of 5.7 months (95% CI 3.1-13.9) compared to 11.2 months (95% CI 6.8-15.6) for patients with SII < 360 (*P* = .087) (HR 1.76, 95%CI 0.91-3.38, *p* = 0.091). SII < 360 showed a higher percentage of response at the first sorafenib re-evaluation than those SII ≥ 360 (24% *vs*. 0%, respectively) (*P* = 0.039) (Table 2).

**Table 2:** Association between SII, NLR and PLR and ORR

	SII (baseline)			NLR (baseline)			PLR (baseline)		
	<360	≥360		<3	≥3		<15.0	≥15.0	
	No. (%)	No. (%)	*P*	No. (%)	No. (%)	*P*	No. (%)	No. (%)	*P*
ORR									
CR+PR	5 (23.8)	0		5 (19.2)	0		5 (15.6)	0	
SD+PD	16 (76.2)	24 (100)	0.039	21 (80.8)	19 (100)	0.063	27 (84.4)	13 (100)	0.301

SII, systemic immune-inflammation index; NLR, neutrophil-to.lymphocyte ratio; PLR, platelet-to-lymphocyte ratio; ORR, objective response rate; CR, complete response; PR, partial response; SD, stable disease; PD, progressive disease

To evaluate SII modifications during the course of treatment. We considered PFS and OS after stratifying patients into 2 groups according to SII levels at baseline and after second blood sample. The first group included patients with high (<360)-high (≥360) levels of SII, while the second included those with high(≥360)-low(<360), low(<360)-low(<360) SII. Patients in the first group had a median PFS of 2.5 months compared to 3.9 months for those in the second group (HR 1.77, 95% CI 0.93–3.36, p=0.08) (Figure 1c). OS was 13.9 months in the first group and 5.2 months in the second group (HR 2.07, 95% CI 1.03–4.13, p=0.040) (Figure 1d).

NLR ≥ 3 was associated with a median PFS of 2.6 months (95% CI 1.7-3.7) compared to 3.3 months (95% CI 2.6-6.2) for patients with NLR < 3 (*P* = .049) (HR 1.84, 95%CI 0.99-3.41, *p* = 0.053) (Figure 1e). NLR ≥ 3 was associated with a median OS of 5.6 months (95% CI 2.2-10.4) compared to 13.9 months (95% CI 5.2-20.9) for patients with NLR< 3 (*P* = .058) (HR 1.87, 95%CI 0.97-3.60, *p* = 0.062) (Figure 1f).

PLR ≥ 15.0 was associated with a median PFS of 2.6 months (95% CI 2.0-5.2) compared to 2.9 months (95% CI 2.6-8.2) for patients with PLR < 0.15 (*P* = .430) (HR 1.30, 95%CI 0.68-2.49, *p* = 0.433) (Figure 1g). PLR < 15.0 was associated with a median OS of 6.9 months (95% CI 5.6-13.9) compared to 14.6 months (95% CI 2.2-10.0) for patients with PLR≥ 15.0 (*P* = .815) (HR 1.09, 95%CI 0.53-2.26, *p* = 0.815) (Figure 1h).

NLR and PLR modifications during the course of treatment show in Table 3.

**Table 3:** NLR and PLR modifications during the course of treatment

	N. pts	PFS			OS		
		N. events	HR (95% CI)	p	N. events	HR (95% CI)	p
**NLR**	48	41	1.08 (0.98-1.20)	0.129	35	1.15 (1.03-1.27)	0.010
**PLR**	49	42	0.98 (0.94-1.02)	0.348	36	1.01 (0.97-1.05)	0.710

The counts for neutrophils, lymphocytes and platelets alone without the ratio and clinical outcome show in Table 4.

**Table 4:** The counts for neutrophils, lymphocytes and platelets alone without the ratio and clinical outcome

	N. pts	N. events	Median PFS (95% CI)	p	HR (95% CI)	p
**Neutrofili**:					1.000 (1.000-1.000)	0.181
<UNL	8	7	3.9 (2.5-11.2)		0.77 (0.34-1.75)	0.533
< >UNL	43	35	2.8 (2.2-3.9)		1.00	
>UNL	5	4	2.3 (1.2-nr)	0.516	1.59 (0.56-4.58)	0.386
**Linfociti:**					1.000 (1.000-1.000)	0.317
≤UNL	13	13	2.6 (1.8-3.9)		1.00	
>UNL	43	33	2.9 (2.5-6.0)	0.287	0.70 (0.37-1.35)	0.291
**Piastrine**:					1.000 (0.996-1.003)	0.811
≤UNL	21	17	3.9 (2.0-8.2)		1.00	

	N. pts	N. events	Median OS (95% CI)	p	HR (95% CI)	p
**Neutrofili**:					1.000 (1.000-1.000)	0.199
<UNL	8	5	13.1 (4.5-nr)		0.57 (0.22-1.47)	0.244
< >UNL	43	31	6.9 (3.7-14.6)		1.00	
>UNL	5	3	6.7 (2.0-nr)	0.419	1.34 (0.40-4.52)	0.639
**Linfociti:**					1.000 (0.999-1.000)	0.307
≤UNL	13	11	5.2 (2.2-19.0)		1.00	
>UNL	43	28	11.2 (5.7-15.6)	0.107	0.56 (0.27-1.15)	0.112
**Piastrine**:					0.999 (0.995-1.002)	0.431
≤UNL	21	15	6.8 (3.1-20.9)		1.00	
>UNL	35	24	6.9 (5.2-14.9)	0.655	1.16 (0.60-2.25)	0.656

After adjusting for clinical covariates (age, gender, etiology, BCLC stage, ECOG performance status), SII and NLR remained an independent prognostic factor for OS (SII: HR=2.99, 95% CI 1.34-6.68, p= 0.007; NLR: HR= 2.36, 95% CI 1.07-5.18, p = 0.033) but not for PFS (HR=1.73, 95% CI 0.91-3.29, p=0.096; NLR: HR=1.81, 95% CI 0.92-3.58, p=0.088).

## DISCUSSION

In the present study, SII and NLR was show to be an indipendent predictor of OS for patients with HCC treated with sorafenib. Our results suggest that the SII could be a more objective marker that reflects the balance between host inflammatory and immune response status than indexes such as the PLR and NLR. In addition, our data have shown that a high SII basal and a month is associated with a worse prognosis respect other patients.

In neoplastic process, inflammatory cells are powerful tumor promoters; they produce an attractive environment for tumor growth, facilitating genomic instability and promoting angiogenesis [19]. Tumors are often infiltrated by various numbers of lymphocytes, macrophages and mast cells. It has been suggested that lymphocytes play central roles in host antitumor immune responses. Mouse models have shown that lymphocytes may control cancer outcome [20].

As an integrated indicator based on peripheral lymphocyte, neutrophil, and platelet counts, the predictive value of SII for cancer outcomes might be due to the function of these three types of cells. Lymphocytes and platelets have been proven to promote tumor development. In addition, recent evidence indicates that neutrophils enhance cancer cell invasion, proliferation, and metastasis and assist cancer cells with evading immune surveillance.

Several studies have shown that platelets induces circulating tumor cell epithelial-mesenchymal transition and promotes extravasation to metastatic sites [21, 22]. Neutrophils promote adhesion and seeding of distant organ sites through the secretion of circulating growth factors such as vascular endothelial growth factor (VEGF) and proteases [23, 24]. Lymphocytes play a crucial role in tumor defense by inducing cytotoxic cell death and inhibiting tumor cell proliferation and migration, thereby dictating the host's immune response to malignancy [25]. Thus, inflammation induces changes in the cancer microenvironment changes that favor cancer progression.

Ipilimumab is a monoclonal antibody that works to activate the immune system by targeting CTLA-4, a protein receptor that downregulates the immune system. Recent works on melanoma have shown thatb derived neutrophil-to-lymphocyte ratio may be associated with response to these drugs [26, 27]. For this reason, our work highlights the possible benefit of a subset of patients with advanced hepatocellular carcinoma to treatment with ipilimumab. In conclusion the low cost, easy determination, and reproducibility of a full blood count make SII and NLR a promising tool for assessing HCC prognosis in future clinical practice.

## PATIENTS AND METHODS

### Patient population

This retrospective study was conducted on 56 HCC patients consecutively treated at our institute (Istituto Scientifico Romagnolo per lo Studio e la Cura dei Tumori) from 2012 to 2015.

We enrolled only patients receiving oral treatment with either 400 mg of sorafenib (consisting of 2 200-mg tablets) twice daily. Treatment with sorafenib was continued until disease progression, unacceptable toxicity or death occurred. Disease progression was assessed using Modified Response Evaluation Criteria in Solid Tumors (mRECIST).

### Statistical analysis

The aim of this analysis was to examine the association between baseline SII, NLR and PLR levels and Progression-Free Survival (PFS) and Overall Survival (OS) in patients with HCC treated with sorafenib.

Information on neutrophil, lymphocyte and platelet counts from hematologic blood tests carried out at baseline (the day before the start of treatment) and one month was collected. Complete blood counts have been carried out with XE-2100 (Sysmex, Kobe, Japan).

The SII was calculated as platelet count × neutrophil count/lymphocyte count, NLR was obtained by dividing the absolute neutrophil count by the absolute lymphocyte count, and the PLR was calculated by as the ratio of the absolute platelet count to the absolute lymphocyte count.

Association between categorical variables was assessed using the Fisher's exact test, when appropriate.

PFS was defined as the time interval between the day of start of treatment and the day of documented disease progression, last follow-up visit if there was no progression or the day of death. OS was defined as the time interval between the day of start of treatment until the day of death or last follow-up visit. PFS and OS were estimated by the Kaplan-Meier method and curves were compared by the log-rank test. Unadjusted and adjusted hazard ratios (HRs) by baseline characteristics (age, gender, etiology, ECOG performance status) were calculated using the Cox proportional hazards model.

We also conducted landmark analyses to reduce possible confounding by time on treatment by assessing the impact of change in SII; NLR and PLR at 1 month landmark time on survival outcomes. X-tile 3.6.1 software (Yale University, New Haven, CT) was used to determine the cutoff value for baseline levels of each II. SII ≥360, NLR ≥3 and PLR ≥15 were considered as elevated levels.

All p values were based on two-sided testing and statistical analyses were performed using SAS statistical software version 9.4 (SAS Inc., Cary, NC, USA).
